# Evaluating the Effectiveness of Mobile Apps on Medication Adherence for Chronic Conditions: Systematic Review and Meta-Analysis

**DOI:** 10.2196/60822

**Published:** 2025-07-31

**Authors:** Vaidehee Lanke, Kevin Trimm, Bettina Habib, Robyn Tamblyn

**Affiliations:** 1 Department of Epidemiology, Biostatistics and Occupational Health, McGill University, 2001 McGill College Ave, Montreal, QC, H3A 1Y7, Canada, 1 3062227521; 2 Department of Experimental Medicine, McGill University, Montreal, QC, Canada

**Keywords:** medication adherence, systematic review, mobile apps, chronic conditions, mHealth, smartphone, chronic disease, digital health, mobile health

## Abstract

**Background:**

Medication adherence is crucial for managing chronic conditions. Mobile apps may have the potential, through a wide variety of features, to support and improve medication adherence.

**Objective:**

The purpose of this systematic review was to evaluate the effectiveness of mobile apps in promoting medication adherence for patients managing chronic conditions.

**Methods:**

MEDLINE (Ovid), Embase (Ovid), and Cochrane Central Register of Controlled Trials databases were searched for randomized controlled trials (RCTs) evaluating the effectiveness of mobile app interventions in improving medication adherence in patients with chronic conditions. Study design and app features were qualitatively described. Meta-analyses were performed on studies, grouped by medication adherence measurement scale, on the mean differences in medication adherence scores between intervention and control groups, using random effects models. If baseline medication adherence data were available, a difference in differences meta-analysis with a random effects model was also conducted. Bias assessment was conducted using the Cochrane Risk of Bias tool.

**Results:**

This review included 14 RCTs published between 2014 and 2022, with sample sizes between 57 and 412 participants and the length of interventions ranging from 30 days to 12 months. A range of patient populations was evaluated, including those with Parkinson disease, coronary heart disease, psoriasis, and hypertension, with hypertension being the most common condition. All 14 studies reported that app interventions improved medication adherence, and 10 RCTs demonstrated statistically significant improvement in medication adherence. Three separate sets of meta-analyses, categorized by the medication adherence measurement scales, were conducted on the mean difference between medication adherence scores between the control and intervention groups: the 8-item Morisky Medication Adherence Scale (MMAS-8; 0.57, 95% CI 0.33-0.80; *P*<.001, *I*^2^=0%, τ^2^=0, *P* value for heterogeneity test=.94), 4-item Morisky Medication Adherence Scale (MMAS-4; 0.15, 95% CI −0.12 to 0.42; *P*=.28, *I*^2^=0%, τ^2^=0, *P* value for heterogeneity test=.54) and a percentage medication adherence scale (18.85, 95% CI 2.17-35.53; *P*=.03, *I*^2^=63%, τ^2^=94.89, *P* value for heterogeneity test=.10). Additionally, with available baseline adherence scores, difference in differences meta-analyses were conducted for studies using the MMAS-8 scale (0.38, 95% CI 0.15-0.62; *P*=.001, *I*^2^=0%, τ^2^=0, *P* value for heterogeneity test=.51) and for studies using the MMAS-4 scale (0.55, 95% CI 0.17 to 0.93; *P*=.005, *I*^2^=33%, τ^2^=0.03, *P* value for heterogeneity test=.22). The meta-analysis of the MMAS-8 scale, percentage medication adherence scale, and both difference-in-differences meta-analyses demonstrated that app-based interventions improved medication adherence.

**Conclusions:**

From the studies included in this review, mobile apps, designed for a wide variety of chronic conditions with a range of features, were shown to improve medication adherence and may be a tool to successfully manage chronic conditions.

## Introduction

The increasing prevalence of chronic illnesses has become a significant public health issue [1,2]. Globally, 1 in 3 adults lives with more than one chronic condition, and in high-income countries, the estimate is closer to 3 in 4 older adults. Significant health, patient, and economic impacts are associated with chronic diseases, and the prevalence of these diseases is increasing substantially [3,4]. While there are a range of types of and treatments for chronic diseases, a common challenge remains the management of complex medication regimens [1], which is needed to slow disease progression, prevent further disease, and reduce the risk of adverse health outcomes [5].

Medications are an important part of the management of chronic diseases [5]. However, the effectiveness of medications depends primarily upon adherence to them [5], defined by the World Health Organization (WHO) as “the extent to which a person’s behavior-taking medications, following a diet and/or executing lifestyle changes–corresponds with agreed recommendations from a health care provider” [6]. While medication adherence is clinically essential for chronic disease management [7], the WHO estimates only 50% of chronically ill patients take medications as prescribed in high-income countries [8]. The causes of medication nonadherence are complex and can include insufficient information about the illness and the use of medications, adverse medication effects, inability to pay for medication, and poor memory [5,8]. The clinical and economic costs of medication nonadherence are immense; for example, in the United States, poor medication adherence causes about 33% to 69% of all medication-related hospitalizations and results in an estimated $100 billion in health care costs per year [9,10].

While many interventions exist to support medication adherence for chronic conditions, such as motivational interviewing and pharmacist-led multidisciplinary education, these interventions are time and labor intensive [11]. There is a need for more practical interventions to improve medication adherence of patients with chronic disease [11].

Mobile apps have the potential to meaningfully support and increase medication adherence [9,12]. With currently more than an estimated 97,000 mobile health (mHealth) apps available on various platforms, the fifth largest category of mHealth apps is aimed at medical condition management, including medication adherence [13]. As of 2017 alone, there was an estimated 10,000 mHealth apps that provided medication reminders [14]. Compared to other digital interventions that target medication adherence, such as electronic medicine boxes that register the date and time the box was opened so that a detailed record of when medications are taken is generated [15], apps, due to the ubiquitous nature of smartphones, are significantly accessible and low-cost medication adherence interventions [9].

Medication adherence apps aim to consolidate the entirety of a user’s medication-specific information in one place and provide essential education about the disease or care [9]. Features of current medication adherence apps include reminders for medication consumption and refills, doses that can be logged, data logs to be accessed by patients or uploaded to care providers, and medication information such as adverse effects or dosages [9]. According to Heldenbrand et al [16], these features can be broadly categorized into general features, adherence attributes, medication management, and connectivity. General features include free apps that are multilingual, compatible with multiple platforms, and are advertisement-free. Adherence attributes include tracking taken and missed doses, specific medication reminders that are customizable, and medical social networking. Medication management features include complex medication instructions, information databases of medication, and the ability to identify potential interactions. Connectivity features, including cloud data storage, ability to export and share data, and the ability to generate reminders without cellular or WiFi connectivity [16].

While the number of apps directed at medication adherence is increasing, there is a need for more research on patient use of these mobile apps, specific app features, and their effect on medication adherence [9,12]. Previous systematic reviews on the impact of mobile apps on medication adherence have predominately covered medication adherence regardless of acute or chronic conditions or focused on one specific condition, such as asthma or cardiovascular disease. Some did not include accompanying meta-analyses, making it difficult to quantify the impact of apps [11,13,17-21]. Additionally, since the number of medication adherence apps is rapidly increasing and advanced technical features are now possible, a review of the most up-to-date evidence is warranted. Therefore, the objective of this systematic review was to investigate the effectiveness of mobile apps in improving medication adherence in randomized controlled trials (RCTs) of patients with chronic conditions.

## Methods

This review followed the PRISMA (Preferred Reporting Items for Systematic Reviews and Meta-Analyses) guidelines for reporting. The review protocol was registered in PROSPERO (International Prospective Register of Systematic Reviews) database (CRD42023488188).

### Search Strategy

MEDLINE (Ovid), Embase (Ovid), and CENTRAL (Cochrane Central Register of Controlled Trials) databases were searched from inception to September 2023. The search strategy was developed by identifying keywords from the research question and relevant studies. The key terms in the search strategy included mobile apps, smartphones, medication adherence, and medication therapy management. The search strategy was first developed for MEDLINE and then adapted for the other databases (all search strategies are presented in MultimediaAppendices 1^3^).

Studies of interest to this review were RCTs that investigated the effectiveness of stand-alone mobile apps in improving medication adherence for those with chronic conditions. The inclusion criteria used to select studies were:

- Participants aged 18 and older with a chronic disease, defined as conditions that last for 3 months or longer, may progressively get worse and usually cannot be cured but controlled, such as cardiovascular diseases, hypertension, diabetes, etc [22].
- An intervention group that was randomized to receive a mobile app designed to promote medication adherence.
- A control group that was randomized to receive usual or standard of care that did not involve any mobile app.
- The outcome, medication adherence, had to be quantitatively reported for both intervention and control groups. Medication adherence could be reported through direct (measurement of level of drug, biological marker in the blood, etc) or indirect (pill counts, self-reporting, etc) measures.
- Articles must be published in English.

The exclusion criteria used to reject studies were:

- Participants had acute conditions such as myocardial infarction or stroke.
- The intervention was not based on a mobile app and only involved SMS text messaging.
- The intervention had components beyond the mobile app, such as counseling with health care professionals.
- The control group received an app-based intervention.
- The only outcome assessed was adherence to components other than medications, such as appointment attendance or laboratory work.

A 2-stage screening process was used to select studies. All studies identified in the 3 databases were compiled in Covidence, a systematic review management web-based platform [23]. Covidence identified and subsequently removed duplicate records. In the first stage of screening, 2 reviewers (VL and KT) independently reviewed the title and abstract of all studies and excluded studies based on the eligibility criteria. In the second stage of screening, 2 reviewers (VL and KT) independently read the full text of studies and selected studies to include in the review based on whether they met the eligibility criteria. To resolve disagreements or uncertainties regarding decisions about study inclusion or exclusion, the same 2 reviewers met to discuss and reach a consensus.

### Data Extraction and Data Synthesis

Data was extracted by one reviewer (VL) using the Cochrane data collection form for interventions. Data extracted included evidence that studies met eligibility criteria, study design and setting, participant characteristics (age, sex, medical illness, severity of illness), intervention and control group interventions, features of the mobile apps, how medication adherence was measured and medication adherence results.

To allow for fair comparisons, identified studies were grouped by the method used to measure medication adherence. Medication adherence scores were compiled and grouped by the following medication adherence measurement methods, 8-item Morisky Medication Adherence Scale (MMAS-8), percentage medication adherence, Hill Bone compliance scales, 4-item Morisky Medication Adherence Scale (MMAS-4), and self-made scale (Multimedia Appendix 4 [24-37]). Although each method measured medication adherence, each method was designed differently and produced a variety of adherence scores. Therefore, the meta-analysis was stratified by adherence measurement method as conducting a single meta-analysis, with all studies included, may not have resulted in a just comparison or have clinical interpretability.

Meta-analyses were conducted in subgroups of studies that used the same method of measuring medication adherence. For each meta-analysis conducted, a random effects model was used, with the measure of effect being the mean difference between intervention and control groups in medication adherence scores at the end of the study period. Additionally, in studies that measured adherence at baseline and at the completion of the study period, a difference-in-differences meta-analysis was conducted. Individuals were the unit of analysis. Since all studies in the review were RCTs, it was assumed that the baseline medication adherence between control and intervention groups was similar. Heterogeneity was assessed using the Cochran Q test; the *I*^2^ statistic to assess the percentage of total variation due to heterogeneity, with *I*^2^ above 50% considered moderate to substantial heterogeneity; and τ^2^ statistic to assess the in-between study variance [38]. The meta package in R (R Core Team) was used to conduct the analysis and create forest plots to display the results [39].

### Quality Assessment

Two reviewers (VL and BH) independently conducted the quality assessment on each included study using the Cochrane Risk of Bias form [40]. The following 7 domains were assessed: random sequence generation, allocation concealment, selective reporting, blinding of participants and personnel, blinding of outcome assessment, incomplete outcome data, and other sources of bias. For each domain, an assessment of low, high, or unclear risk of bias was made. To discuss the assessments and resolve disagreements, the same 2 reviewers met and reached a consensus on the bias assessment.

## Results

### Search Results

**Figure 1.**
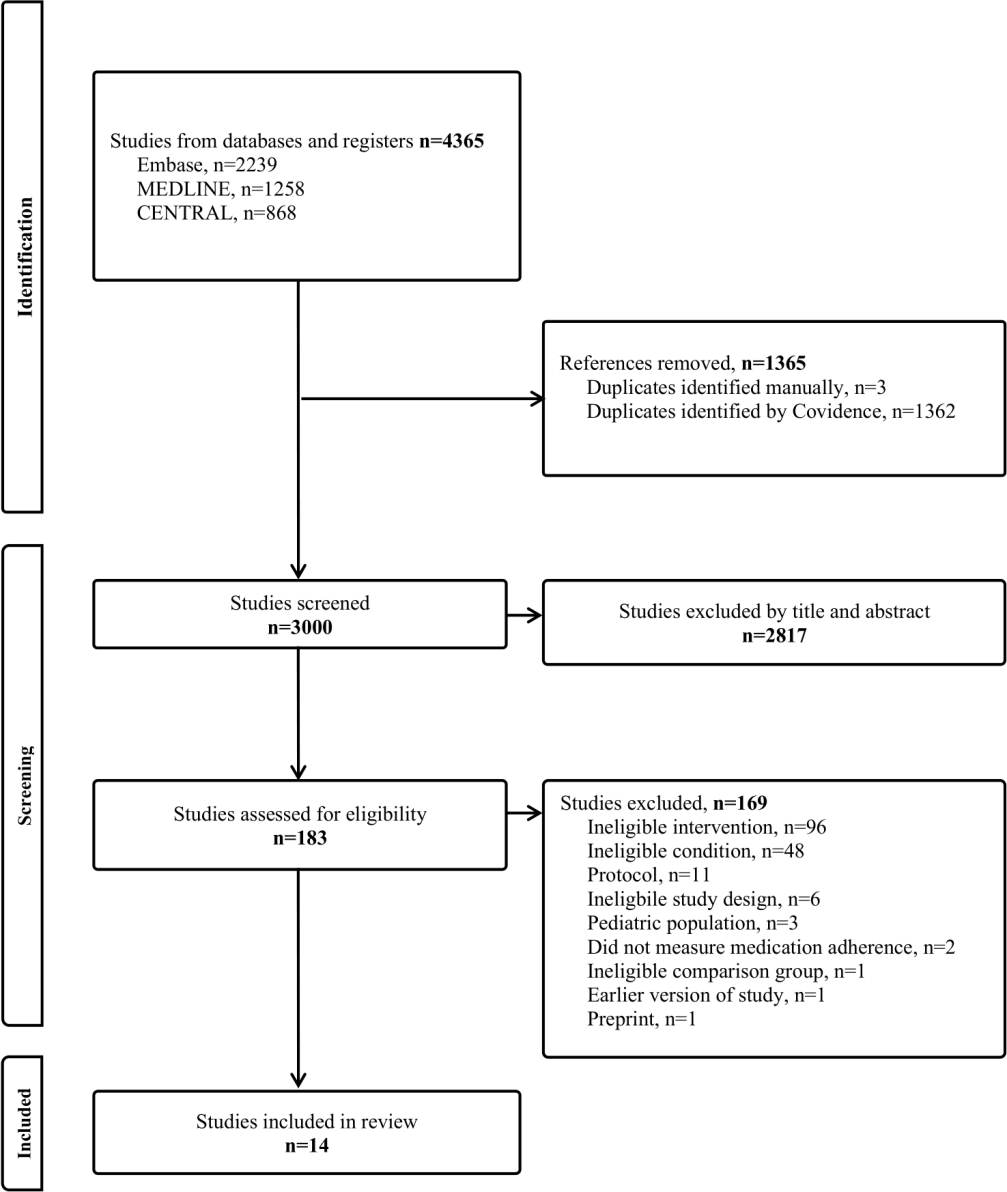
PRISMA (Preferred Reporting Items for Systematic Reviews and Meta-Analyses) flow diagram summarizing study search and selection process.

The search from the 3 databases identified 4365 studies, of which 1365 were duplicates and removed. The remaining 3000 studies were screened by title and abstract, with 2817 studies excluded because they did not meet the eligibility criteria. Out of the 183 studies assessed by full text for inclusion in the review, 169 were excluded for reasons including nonapp intervention, acute condition, study design, and comparison group. Therefore, 14 studies were included in this review. The search and study selection process is summarized in a PRISMA flow diagram (Figure 1).

### Study Characteristics

The studies included in this review were published between 2014 to 2022. The sample size varied from 57 [24] to 412 participants [25] and the length of intervention varied from 30 days [24,26] to 12 months [27] (Table 1). Of the 14 studies, 13 were parallel 2-arm RCTs that compared a mobile app intervention for medication adherence with a control group [24-36]. The remaining study was a parallel 3-arm RCT that compared 2 versions of an app, one with basic and one with more advanced features, with a control intervention [37]. In this study, only the basic app met the inclusion criteria, and the results for its comparison with the control group were used in the analysis.

**Table 1.** Characteristics of the selected studies.

Source and country	RCT^a^ design	Condition	Total participants randomized, n	Average age (years), n	Length of intervention	Participants recruited from	Intervention arm	Control arm
Abu-El-Noor et al [28]; Palestine	Parallel 2-arm	Hypertension	218	56.5	3 months	Primary health care centers	Mobile app	Usual care: participants continued their daily routine
Bozorgi et al [29]; Iran	Parallel 2-arm	Hypertension	120	51.8	8 weeks	Tertiary medical center	Mobile app	Usual care: participants’ medical history was taken, underwent physical examinations (eg, measurement of blood pressure and weight) and laboratory tests, were offered paraclinical services tailored to the individuals’ conditions, and provided drug treatments according to the JNC8 recommendations
Guhl et al [26]; United States	Parallel 2-arm	Atrial fibrillation	120	72.1	30 days	Ambulatory facilities at a medical center	Mobile app	Usual care
Hammonds et al [24]; United States	Parallel 2-arm	Depression	57	20.6	30 days	Campus-based advertisement, flyers, and university recruitment system	Mobile app	Usual care: participants were instructed to continue to take medications as prescribed by their physician
Horvath et al [30]; United States	Parallel 2-arm	HIV ART^b^	90	37.4	4 months	Grindr, flyers, and palm cards at community-based organizations and clinics that served the targeted population.	Mobile app	No treatment
Lakshminarayana et al [31]; United Kingdom	Parallel 2-arm	Parkinson disease	215	60.3	16 weeks	Health centers	Mobile app	Usual care: participants, at the 16-week appointment, had regular outpatient clinical assessments conducted, including symptom and medication reviews
Márquez Contreras et al [27]; Spain	Parallel 2-arm	Hypertension	154	57.4	12 months	Primary health care centers	Mobile app	Usual care: participants received usual care for high blood pressure including control every 6 months of blood pressure, annual control of therapeutic adherence, annual analysis, and biannual electrocardiogram
Mira et al [32]; Spain	Parallel 2-arm	Multiple chronic conditions	102	71.9	3 months	Health centers	Tablet app	No intervention
Morawski et al [25]; United States	Parallel 2-arm	Hypertension	412	52.05	12 weeks	Online patient communities, social media, pertinent apps, and targeted advertisement	Mobile app	No intervention
Osahon et al [33]; Nigeria	Parallel 2-arm	Glaucoma	200	52.1	2 months	Out-patient pharmacy	Mobile app	No intervention
Santo et al [37]; Australia	Parallel 3-arm	Coronary heart disease	166	57.9	3 months	Hospital	Basic app: provided simple daily reminders to take medication at correct time; advanced app: additional interactive and customizable features including medication refill reminders, adherence statistics, and interactive daily reminders, etc	Usual care: participants received standard care, determined by their doctors, and included cardiovascular medications, lifestyle advice, and referral to cardiac rehabilitation
Svendsen et al [34]; Denmark	Parallel 2-arm	Psoriasis	134	48.0	4 weeks	Outpatient clinic, advertisement	Mobile app	Usual care: participants received once-daily medication of Cal/BD cutaneous foam, delivered in canisters with dispensers containing an electronic monitor that registered the day and time the dispenser was used. The canister was replaced when empty
Teong et al [35]; Malaysia	Parallel 2-arm	Hyperphosphatemia	74	48.3	12 weeks	Health centers	Mobile app	Usual care: participants received usual medical care from their hemodialysis centers. Participants also received, at baseline, one 30‐ to 40-minute nutrition counseling session with a dietitian on hyperphosphatemia management covering hyperphosphatemia, dialysis removal of phosphorus, sources of dietary phosphorus (eg, animal, plant, and inorganic) with examples of foods high and low in phosphorus content, and the use of phosphate binders. Topics were accessible to the participant in a 12-page illustrated booklet available in English, Malay, and Mandarin
Torkabad et al [36]; Iran	Parallel 2-arm	Hypertension	78	46.6	3 months	Research center	Mobile app	Usual care

a RCT: randomized controlled trial.

b ART: antiretroviral therapy.

### Participant Characteristics

A range of chronic conditions were considered in this review, the most prevalent being hypertension, where 5 [25,27-29,36] out of 14 of the included studies targeted this condition (Table 1). The other chronic conditions studied included atrial fibrillation, coronary heart disease, psoriasis, and a combination of chronic conditions. The mean age of participants ranged from 20.6 (SD 4.3) years [24] to 72.1 (SD 9.1) years [26]. Studies recruited participants from a range of facilities including primary health care centers [27], a tertiary medical center, and ambulatory facilities at a medical center [26].

### Intervention and Control Arms

None of the control interventions involved a mobile app (Table 1). In 4 out of 14 studies, the control group received no form of alternative intervention [25,30,32,33]. In the remaining 10 studies, control groups received various alternative interventions [24,26-29,31,34-37], including instructed to take medications prescribed by their physician [24]; nutrition counseling sessions with a dietitian on hyperphosphatemia management [35], and received cardiovascular medication, lifestyle advice, and referral to cardiac rehabilitation in 1 study [37].

### Medication Adherence App Features

Overall, the apps differed in the number and type of features they offered (Table 2). Apps were designed to support medication adherence for a specific chronic condition or designed to support medication adherence in general. Of the 14 apps examined, 9 were specifically designed for a certain condition [25-31,34,35], with 4 tailored for participants with hypertension [25,27-29]. A total of 6 of these condition-specific apps featured educational content about the condition and how to manage it [26,28-31,35], 4 allowed users to record and save information about their condition (eg, recording blood pressure) [25,27-29], 2 allowed users to track self-monitoring measures like sleep, exercise, itching, and pain [31,34], and 2 generated reports of user-inputted information such as color-coded graphs of how many doses were taken in the past and current weeks [30,31]. The remaining 5 apps were general medication reminder apps, not condition-specific [24,32,33,36,37] and could be used for any chronic condition.

**Table 2.** Features of the mobile apps from the included studies.

Source	Condition	App features
Abu-El-Noor et al [28]	Hypertension	The mobile app sent daily medication reminder alarms, corresponding to the number and time of prescribed doses, provided daily educational messages about hypertension, treatment, diet therapy, and complications and a monthly reminder message for the next follow-up appointment, and allowed participants to record blood pressure readings to show health care providers.
Bozorgi et al [29]	Hypertension	The Blood Pressure Management app allowed participants to record and save blood pressure, plotted blood pressure in a chart, received feedback on recorded blood pressure, provided reminders for time of drug consumption, next appointment date and blood pressure measurement, provided information about healthy diet, weight loss plans and the nature, control and treatment of the disease, suggested supportive programs for smoking cessation, sent notifications to patient’s family members of critical blood pressure levels, sent general and specific, based on patient characteristics, motivational messages and reminders about treatment adherence, and allowed participants to save health information on a portal for physicians and researchers.
Guhl et al [26]	Atrial fibrillation	The mobile app featured Tanya, a smartphone-based relational agent, that simulated face-to-face conversations with a health coach using speech and animated behavior. Tanya provided health and atrial fibrillation education, symptoms, monitoring, adherence, and problem-solving for users, according to participant needs and was tailored to each user (ex. used participant name during interactions). The app referred users to the Kardia regularly to encourage use, provided instructions on how to use the device, and directed users to check rhythm concomitant with reporting symptoms.
Hammonds et al [24]	Depression	The medication reminder app provided reminders for medications and information about medications (prescriptions, doses, etc) and participants indicated whether they had taken their medication by responding to a message received by the app.
Horvath et al [30]	HIV ART	The APP+, a mobile app, had 3 main components. One, it provided users a homepage with 5 new pieces of HIV or ART adherence content each day. The content was intended to address the informational, motivational, and behavioral skills by discussing adherence side effects, adherence and mental health, motivational messages to adhere, adherence strategies, general issues of living with HIV, and tips on how to use the APP+ mobileapp. Second, a story of a fictional character living with HIV, using substances and being sexually active was presented to the user to provide specific medication adherence tips. Each installment of the storyline was available every 6 hours after users’ response and asked participants to choose options for how to proceed and involved scenarios such as mixing drugs and HIV medications, medication side effects, and not having HIV medications easily available. Third, the “My Meds” tab allowed users to self-monitor medication adherence: reminders appeared for each scheduled dose, participants reported whether they took the dose or skipped it, and generated color-coded graphs of how many doses were reported taken in the current and past weeks.
Lakshminarayana et al [31]	Parkinson disease	The Parkinson Tracker app allowed participants to track 10 self-monitoring measures on a 5-point scale including sleep, exercise, mood, energy, movement, and suppleness, had a reminder system with alerts to help participants track medications, generated a data report of information entered by participant over trial period to aid follow-up appointment, had games to track physical responsiveness and cognition and provided information about Parkinson disease from Parkinson’s UK and the Cure Parkinson’s Trust.
Márquez Contreras et al [27]	Hypertension	AlerHTA, a mobile app, allowed participants to record personal data and doctor’s guidance about prescribed treatment and posology, set recommended blood pressure levels as objectives, and provided reminder alarms for medications or events and recorded results of blood pressure measurement.
Mira et al [32]	Multiple chronic conditions	ALICE, a medication tablet-based self-management app, stored details and images of prescriptions, related medication instructions and doctors' recommendations, had a customized system of reminders to alert patients when to take medication, along with information about medication (name, dose, time, warnings) and enabled monitoring of adherence to prescriptions and medical advice via wireless connection to the study monitoring system, the health care provider, and a caregiver if authorized by the patient. The app sent the complete list of prescriptions to caregivers, along with a summary of patients’ adherence behavior.
Morawski et al [25]	Hypertension	The Medisafe app allowed patients to enter medication lists manually along with the preferred time of administration or autopopulate medications through linkage with an existing medication record, provided alerts to remind time to take medications, generated weekly adherence reports, allowed patients to track blood pressure and other biometric measurements, and allowed users to assign a “Medfriend” who was granted access to the patient’s medication taking history, received alerts when doses were missed, and could provide peer support.
Osahon et al [33]	Glaucoma	medPlan was a medication reminder mobile app designed to promote medication adherence.
Santo et al [37]	Coronary heart disease	The medication reminder app allowed users to store their current list of cardiovascular medications and set and receive daily reminders at the time medication is to be taken.
Svendsen et al [34]	Psoriasis	The medication reminder app provided daily information on amount of treatment and number of treatment apps, allowed patients to rate their symptoms (itching, pain, inflammation, dryness, stress, etc) on an interval scale, and provided daily treatment reminders.
Teong et al [35]	Hyperphosphatemia	MyKidneyDiet-Phosphate Tracker, a mobile app in 3 languages, English, Malay, and Mandarin, provided 6 animated education videos on hyperphosphatemia, dialysis, phosphate binders, dietary phosphorus, lifestyles, and the responsibilities of a dialysis patient. The app also had an interactive food database of more than 500 foods commonly consumed by patients with hyperphosphatemia in Klang Valley, Malaysia, and had a personalized diet calculator that calculated the required phosphate binder dose, titrated to the phosphate content of foods chosen for meals by the patient.
Torkabad et al [36]	Hypertension	DaroYab 2.1.0, a mobile app, allowed users to record name of the drug, medication dose, precautions, and time (date, day, hour) medication is to be taken, reminded participants by voice alarm and text message to take medication, and provided drug information on generic drugs, herbal medicines, and their side effects.

Out of the 14 apps, 12 had explicit medication reminder functions [24,25,27-34,36,37]. These apps sent medication reminder alerts at the appropriate time and according to the number of prescribed doses. Of the remaining 2 apps, one featured a smartphone-based relational agent that simulated face-to-face conversations and provided education regarding medication adherence [26] and the second app had educational video content, including on lifestyles and responsibilities of being a dialysis patient [35]. Some apps involved users’ caregivers by sending notifications to them about critical health information like blood pressure levels, sharing monitoring of medication adherence to prescriptions, or granting access to a patient’s medication taking history, including receiving alerts when medication doses were missed [25,29,32].

Of the 14 apps, 9 had medication-specific information available [24,26,28-30,32,34-36], for example, daily educational messages about hypertension [28] and animated education videos on hyperphosphatemia, dialysis, and phosphate binders [35]. Six out of 14 apps had a component of education [26,28-31,35], including information about atrial fibrillation [26] and Parkinson disease from Parkinson’s UK and the Cure Parkinson’s Trust.

Out of the 14 apps, 3 used unique methods of user engagement [26,30,35]. In one study, a smartphone-based relation agent, Tanya, was used that simulated in-person conversations with a health coach and provided information on atrial fibrillation, its symptoms, monitoring, and medication adherence [26]. Another study used interactive storytelling where users were invited to choose what happens next to a fictional character experiencing similar health conditions as them. This approach was used to provide users with specific medication adherence tips [30]. Finally, an app meant to support patients with hyperphosphatemia had a personalized diet calculator function [35]. The interactive database used in this app enabled meal calculations for elements such as energy, protein, and the required phosphate binder dose for each meal.

### Assessment of Adherence

A total of 13 of 14 studies used continuous medication adherence measures. These medication adherence measures can be categorized into 5 categories: Hill Bone compliance scale [28,29], percentage adherent [24,27,30,34], MMAS-8 [25,31,36,37], MMAS-4 [32,35], and a self-made scale [33] (medication adherence scales, measurements, and results are presented in Multimedia Appendix 4).

The remaining study used dichotomous medication adherence measures. Participants were asked: “Do you sometimes forget to take [name of prescribed anticoagulant medication]?” and “Over the past two weeks, were there days that you did not take [name of prescribed anticoagulant medication]?” The number of participants who answered yes to either question at baseline and at follow-up was used to measure nonadherence between the control and intervention groups [26].

The Hill Bone compliance scale, MMAS-8 and MMAS-4, and self-made scale were self-reported measurements. The percentage adherence was calculated using methods such as pill counts [24] and electronic monitoring devices [27,34].

### Effect of Apps on Medication Adherence

All 14 studies reported that the app improved medication adherence [24-37]. Overall, 10 trials showed statistically significant improvement in medication adherence [25,27-31,33,34,36,37]. In the remaining 4 trials, 2 studies did not demonstrate a significant difference between comparison groups [24,35], 1 study was unclear [32], and in 1 study, the first medication adherence measure was significant, but the second measure was not [26].

**Figure 2.**
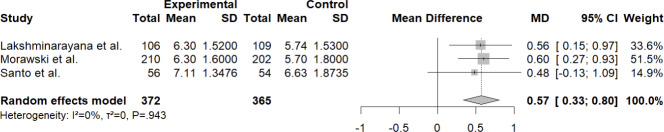
Meta-analysis results of the effect of app interventions on medication adherence for studies that used the 8-item Morisky Medication Adherence Scale. The gray boxes and black lines represent mean differences (MDs) and 95% CIs, respectively. The gray diamond represents the combined MD estimate for all studies, its width signifying 95% CI bounds [25,31,37].

Studies that assessed medication adherence with continuous measures and used the same scales were assessed in the following 3 separate meta-analyses. Mean differences in medication adherence scores at the end of the study period were conducted on studies using the MMAS-8 [25,31,37], MMAS-4 [32,35], and percentage medication adherence [27,30] scales (Figures2^4^).

**Figure 3.**
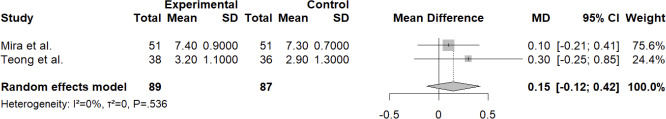
Meta-analysis results of the effect of app interventions on medication adherence for studies that used the 4-item Morisky Medication Adherence Scale [32,35]. MD: mean difference.

**Figure 4.**
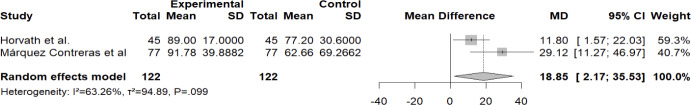
Meta-analysis results of the effect of app interventions on medication adherence for studies that used percentage adherence scale [27,30]. MD: mean difference.

For the MMAS-8 scale, the mean difference between intervention and control groups was 0.57 (95% CI 0.33-0.80; *P*<.001, *I*^2^=0%, τ^2^=0, *P* value for heterogeneity tes =.94). For studies that measured adherence using the MMAS-4 scale, the mean difference between the control and intervention groups was 0.15 (95% CI −0.12 to 0.42; *P*=.28, *I*^2^=0%, τ^2^=0, *P* value for heterogeneity test=.54). For studies using the percentage medication adherence scale, the mean difference in adherence was 18.85 (95% CI 2.17-35.53; *P*=.03, *I*^2^=63%, τ^2^=94.89, *P* value for heterogeneity test=.10). Considering the heterogeneity measures, no statistical heterogeneity was observed in the MMAS-8 and MMAS-4 meta-analyses, therefore increasing the reliability of these meta-analyses results. However, for the percentage medication adherence meta-analysis, the heterogeneity measures indicate moderate to high statistical heterogeneity and suggest variability between the 2 [27,30] studies, perhaps due to study design or lack of standardization of adherence measurement tools, which should be considered when interpreting these findings.

**Figure 5.**
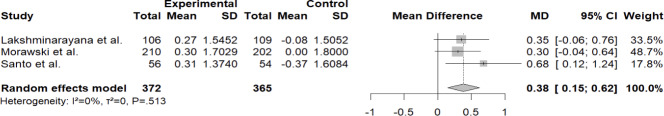
Difference-in-differences meta-analysis results of the effect of app interventions on medication adherence for studies that used the 8-item Morisky Medication Adherence Scale. The gray boxes and black lines represent mean differences (MDs) and 95% CIs, respectively. The gray diamond represents the combined MD estimate for all studies, its width signifying 95% CI bounds [25,31,37].

Additionally, difference-in-differences meta-analyses were conducted, based on adherence scale, for studies that measured adherence before and after the intervention. One difference-in-differences meta-analysis was done for studies using the MMAS-8 scale [24,31,37], and the second meta-analysis was conducted for the studies using the MMAS-4 scale [32,35] (Figures5  6). Both meta-analyses demonstrated that the app intervention increased medication adherence. For studies using the MMAS-8 scale, the mean difference was 0.38 (95% CI 0.15-0.62; *P*=.001, *I*^2^=0%, τ^2^=0, *P* value for heterogeneity test=.51), with no statistical heterogeneity observed. For studies using the MMAS-4 scale, the mean difference was 0.55 (95% CI 0.17-0.93; *P*=.005, *I*^2^=33%, τ^2^=0.03, *P* value for heterogeneity test=.22), with low statistical heterogeneity. The overall low statistical heterogeneity increases reliability in the meta-analyses results and suggests mobile apps can effectively improve medication adherence, even when within-group differences are accounted for.

**Figure 6.**
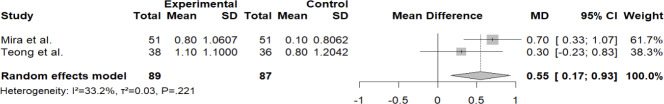
Difference-in-differences meta-analysis results of the effect of app interventions on medication adherence for studies that used the 4-item Morisky Medication Adherence Scale [32,35]. MD: mean difference.

### Risk of Bias

**Figure 7.**
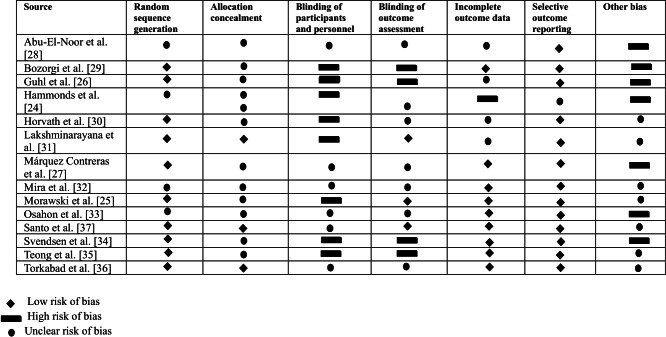
Risk-of-bias assessment on 7 factors for each trial. By symbol, the diamond is low risk of bias, the rectangle is high risk of bias, and the circle is unclear risk of bias [24-37].

In total, 10 out of the 14 studies reported the randomization process adequately [25-27,29-31,34-37] and only 3 studies reported the approach to allocation concealment [25,30,31] (Figure 7) While these types of interventions are difficult to blind, only 3 studies had sufficient outcome assessment blinding [25,31,37]. A total of 9 studies had low risk of incomplete outcome data [25,27,29,32-37] and 13 studies had low risk of selective outcome reporting [25-37]. Finally, 7 trials had an unclear risk for other biases [25,30-32,35-37].

## Discussion

### Principal Findings

A systematic review of 14 RCTs was conducted to evaluate the effectiveness of mobile apps on medication adherence in managing chronic conditions [24-37]. While the number and type of features of each app differed, 12 had explicit medication reminder functions [24,25,27-34,36,37]. Overall, 9 of the 14 apps examined were specifically designed for a chronic condition [25-31,34,35], with 4 of them being designed for participants with hypertension [25,27-29]. The remaining 5 apps were general medication reminder apps applied to chronic conditions [24,32,33,36,37].

Out of the 14 RCTs, 10 trials reported that app-based interventions significantly improved medication adherence [25,27-31,33,34,36,37]. Meta-analyses on the mean difference in medication adherence scores between intervention and control groups for studies using the MMAS-8 scale, percentage medication adherence scale, and both sets of difference in differences meta-analyses indicated that app-based interventions can improve medication adherence [25,27,30-32,35,37], with varied statistical heterogeneity and significance. The heterogeneity among studies should be interpreted cautiously because of the small number of studies used for each meta-analysis [41].

The results of this review are consistent with previous studies in this field that investigated the use of mobile apps on medication adherence. Previous systematic reviews have shown apps to be effective in increasing medication adherence in specific chronic conditions like cardiovascular disease [20] and hypertension [18,42], other chronic conditions [11], diabetes [43], and general conditions [19,21]. The comparability with other systematic reviews suggests that this review contributes to the growing body of evidence supporting app-based medication adherence interventions.

This review can be compared with other types of interventions for medication adherence. Educational interventions are one of the most well-used methods to improve medication adherence with varying effectiveness. For example, a systematic review and meta-analysis of 12 studies found verbal educational interventions improved medication adherence, with low to moderate quality evidence, in patients with hypertension [44]. The average duration of follow-up was 6 months, and a small statistically significant overall effect on medication adherence was found (Cohen *d*=0.18, 95% CI 0.01‐0.34; *P*<.04). Hypertension was the most prevalent condition in this review, and comparatively, our review found that apps had a greater effect on medication adherence, with less follow-up, and the average length of studies in this review was 3 months. For example, in the meta-analysis for studies using the MMAS-8 scale, the mean difference was 0.57 (95% CI 0.33-0.80, *I*^2^=0%, τ^2^=0, *P*=.94) and the difference-in-difference analysis was 0.38 (95% CI 0.15-0.62, *I*^2^=0%, τ^2^=0, *P*=.51).

Another systematic review and meta-analysis of 6 RCTs found education programs had no significant effect at 1 month post-intervention, but significantly improved medication adherence among patients with coronary artery disease at 2 to 6 months post-intervention with moderate evidence (standardized mean difference=1.13, 95% CI 0.33‐1.94; *P*=.006) [45]. While the effect size at the 2- to 6-month mark was larger than the results of our review, the follow-up period was longer than the average length in our review and limited to only one population of patients, whereas our review included several chronic conditions. Compared to these reviews on educational interventions, the results of this systematic review demonstrated that mobile app interventions have consistent and moderate improvements in medication adherence, across a diverse range of chronic conditions in a smaller time span. Additionally, in comparison to the resource-intensive nature of education interventions, mobile apps can be less costly, more accessible, and scalable tools that can be used alongside other medication adherence initiatives such as education programs.

### Limitations

From the results of the Cochrane Risk of Bias assessment, we can infer that the studies used in this review are of moderate quality. The bias assessment highlights blinding of participants, personnel, and outcome accessors and other biases, such as sample size, unvalidated medication adherence measures, and recruitment techniques as the main concerns.

While this review purposely focused on all chronic conditions, the diversity of studies made it challenging to pool data together. For example, the age of participants in included studies ranges from 20.6 years to 72.1 years [24,26]. However, there are differences in mobile health app use by age. Younger populations, commonly defined as under 35 years old, are shown to use apps more frequently than older populations who are not as comfortable using apps [46]. The differences in mobile app comfort and use between different age groups may mean apps impact medication adherence differentially by age, which is not captured by this review.

Another limitation was the wide variation in control interventions. Out of 14 studies, in 4 trials, the control group received no alternative interventions [25,30,32,33], while the remaining 10 control groups received some form of alternative interventions, including different elements from physical examinations to nutrition counseling sessions [24,26-29,31,34-37]. These differences between the control arm of trials make it difficult to compare results across studies because the comparison groups are not the same. Furthermore, the length of intervention ranged from 30 days [24,26] to 12 months [27]. This range makes it difficult to comment on the optimal duration of app use to obtain increased medication adherence. However, since chronic conditions affect different populations and have various treatments, these differences were anticipated, thereby allowing for a review to summarize effectiveness of mobile apps in managing medication adherence for chronic conditions.

Since there is no standardized way to measure medication adherence [47-49], a range of methods were used by studies included in this review, such as self-reporting with different measurement scales, pill counts, and electronic monitoring. This made it difficult to pool data together to perform one meta-analysis to obtain a single summary measure of medication adherence. Instead, multiple meta-analyses based on medication adherence measure type were conducted, and each demonstrated that mobile apps are effective in promoting medication adherence.

Finally, 11 out of 14 studies used self-reporting medication adherence measures [25,26,28-33,35-37]. These measures have different questions, responses, and recall periods, can be subject to social desirability and memory biases, and tend to overestimate medication adherence compared with other assessment methods [50]. Since most of the studies analyzed used self-reported measures, the accuracy of the medication adherence measures might be of concern. However, self-reported measures are the most commonly used instruments to measure medication adherence in the clinical and research settings and are preferred for their speed, efficiency, and low resource cost [50,51].

### Future Research

Future large-scale studies with standardized medication adherence measures are needed to understand the effectiveness of apps in improving medication adherence for chronic conditions.

Future systematic reviews could investigate the impact of specific app features. Since all studies concluded that mHealth apps improved medication adherence, it would be interesting to investigate which app features had the most impact and whether there was a difference between apps with more features compared to simple reminder ones. Similarly, the 14 RCTs had a range of length of intervention, from 30 days to 12 months. It could be investigated what the minimal time and frequency of app usage is to change medication adherence. Another interesting avenue for future research is to consider studies with mobile apps implemented in clinical practice and assess their impact on medication adherence.

Finally, future systematic reviews could investigate the effectiveness of mHealth apps for medication adherence from a patient perspective. To address this, there are a growing number of studies investigating the patient perspective through qualitative methods, such as focus groups. For example, to investigate primary care patients’ comfort sharing health information by mobile device, their awareness, and the use of patient portals, 918 patients completed a survey [52]. It was found that patients were more comfortable sharing mHealth information with providers than having third parties store their information, with patients older than 55 years less comfortable sharing information with providers. Studies, like this one, that investigated patient perspectives on medication adherence apps and their effectiveness could be used in the review, with the summarized knowledge being used to develop more effective adherence apps or when evaluating app effectiveness.

### Conclusion

This systematic review demonstrated that mobile app use is associated with higher medication adherence levels, indicating that apps can improve medication adherence for chronic conditions. The apps investigated in this review had a range of features, from simple reminder notifications to symptom trackers to a smartphone-based relation agent, demonstrating that a variety of app features can support medication adherence. There are limitations to this review, including range of effect sizes between studies, varied control arms, and self-reported medication adherence measures. More evidence, including larger scale trials with standardized methods of measuring medication adherence, is needed to strongly conclude that mobile apps can significantly increase medication adherence for chronic conditions.

## Supplementary material

10.2196/60822 Multimedia Appendix 1 Search strategy: Ovid MEDLINE (1946 to September 2023).

10.2196/60822 Multimedia Appendix 2 Search strategy: Ovid Embase (1946 to September 2023).

10.2196/60822 Multimedia Appendix 3 Search strategy: Cochrane Central Register of Controlled Trials (CENTRAL, on September 13, 2023).

10.2196/60822 Multimedia Appendix 4 Medication adherence scales, measurements, and results from selected studies.

10.2196/60822 Checklist 1 PRISMA (Preferred Reporting Items for Systematic Reviews and Meta-Analyses) checklist.

## Abbreviations

mHealth: mobile health
MMAS-4: 4-item Morisky Medication Adherence Scale
MMAS-8: 8-item Morisky Medication Adherence Scale
PRISMA: Preferred Reporting Items for Systematic Reviews and Meta-Analyses
RCT: randomized controlled trial
WHO: World Health Organization
