# Supplementary material for: Evaluating the Effectiveness of Mobile Apps on Medication Adherence for Chronic Conditions: Systematic Review and Meta-Analysis
Source: J Med Internet Res. 2025 Jul 31;27:e60822. doi: 10.2196/60822 (PMC12312993; doi:10.2196/60822)
Supplement: Multimedia Appendix 1 [file jmir-v27-e60822-s001.doc]

Ovid MEDLINE(R) ALL <1946 to September 12, 2023>

1 Mobile Applications/ 11688

2 Mobile app*.mp. 17906

3 Cell Phone/ 10040

4 Smartphone/ 9267

5 Medication management.mp. 4677

6 Medication Adherence/ 24338

7 Medication Adherence.mp. 32203

8 Medication Therapy Management/ 2782

9 Medication Therapy Management.mp. 3466

10 5 or 6 or 7 or 8 or 9 38735

11 Cell Phone*.mp. 12424

12 Smart Phone*.mp. 1706

13 Smartphone*.mp. 25308

14 Cellphone*.mp. 689

15 Mobile phone*.mp. 14298

16 1 or 2 or 3 or 4 or 11 or 12 or 13 or 14 or 15 55043

17 10 and 16 1258
