# Supplementary material for: Evaluating the Effectiveness of Mobile Apps on Medication Adherence for Chronic Conditions: Systematic Review and Meta-Analysis
Source: J Med Internet Res. 2025 Jul 31;27:e60822. doi: 10.2196/60822 (PMC12312993; doi:10.2196/60822)
Supplement: Multimedia Appendix 2 [file jmir-v27-e60822-s002.doc]

Embase Classic+Embase <1947 to 2023 September 12>

1 mobile application/ 21960

2 mobile app*.mp. 25096

3 mobile phone/ 22366

4 mobile phone*.mp. 29003

5 Cell phone*.mp. 6256

6 smartphone/ 26513

7 Smartphone*.mp. 36812

8 smart phone*.mp. 3549

9 1 or 2 or 3 or 4 or 5 or 6 or 7 or 8 78212

10 medication therapy management/ 15041

11 medication therapy management.mp. 15620

12 Medication management.mp. 7730

13 medication compliance/ 46719

14 Medication adherence.mp. 25485

15 10 or 11 or 12 or 13 or 14 72993

16 cellphone*.mp. 1004

17 9 or 16 78568

18 15 and 17 2239
