# Supplementary material for: Evaluating the Effectiveness of Mobile Apps on Medication Adherence for Chronic Conditions: Systematic Review and Meta-Analysis
Source: J Med Internet Res. 2025 Jul 31;27:e60822. doi: 10.2196/60822 (PMC12312993; doi:10.2196/60822)
Supplement: Multimedia Appendix 3 [file jmir-v27-e60822-s003.doc]

Search conducted on September 13

ID Search

#1 [mh ^"Mobile Applications"]

#2 ("Mobile" NEAR/2 app*):ti,ab,kw

#3 [mh ^"Cell Phone"]

#4 [mh ^Smartphone]

#5 "Medication management":ti,ab,kw

#6 [mh ^"Medication Adherence"]

#7 "Medication Adherence":ti,ab,kw

#8 [mh ^"Medication Therapy Management"]

#9 "Medication Therapy Management":ti,ab,kw

#10 #5 OR #6 OR #7 OR #8 OR #9

#11 ("Cell" NEAR/2 Phone*):ti,ab,kw

#12 ("Smart" NEAR/2 Phone*):ti,ab,kw

#13 Smartphone*:ti,ab,kw

#14 Cellphone*:ti,ab,kw

#15 ("Mobile" NEAR/2 phone*):ti,ab,kw

#16 #1 OR #2 OR #3 OR #4 OR #11 OR #12 OR #13 OR #14 OR #15

#17 #10 AND #16
