# Supplementary material for: Evaluating the Effectiveness of Mobile Apps on Medication Adherence for Chronic Conditions: Systematic Review and Meta-Analysis
Source: J Med Internet Res. 2025 Jul 31;27:e60822. doi: 10.2196/60822 (PMC12312993; doi:10.2196/60822)
Supplement: Multimedia Appendix 4 [file jmir-v27-e60822-s004.doc]

**Table S1.**

| Source | Medication adherence scales | Measurements | Results |
| --- | --- | --- | --- |
| Abu-El-Noor et al. [28] | Measured by a modified 15-item Hill-Bone compliance to high blood pressure therapy scale. Each item was measured on a four-point Likert scale with the corresponding points: never (1), occasionally (2), often (3) and always (4). The possible scores ranged from a minimum of 15 to a maximum of 60 points, with lower scores indicating higher adherence to treatment. | Means of the Hill–Bone compliance to high blood pressure therapy scale for control and interventions groups at baseline and end of follow-up. | Mean (SD)  Intervention  Baseline: 15.64 (4.59)  3 months: 11.73 (3.57)  Control  Baseline: 15.92 (4.48)  3 months: 13.98 (3.70) |
| Bozorgi et al. [29] | Measured by the 14-item Hill-Bone scale. The possible scores ranged from 14 to 70. | Means found from the Hill-Bone checklist for control and intervention groups at baseline and end of follow-up. | Mean (SD)  Intervention  Baseline: 58.5 (7.42)  8 weeks: 65.1 (2.44)  Control  Baseline: 59.1 (5.07)  8 weeks: 59.7 (3.69) |
| Guhl et al. [26] | Measured by asking participants (1) “Do you sometimes forget to take [name of prescribed anticoagulant medication]?” and (2) “Over the past two weeks, were there days that you did not take [name of prescribed anticoagulant medication]?” | Number of yes counts to the self-reported adherence questions for control and intervention groups at baseline and end of follow-up. | N (%) of participants answering “yes” to each question  Question 1  Intervention  Baseline: 17 (27.9)  30 days: 2 (3.5)  Control  Baseline: 13 (22)  30 days: 13 (23.2)  Question 2  Intervention  Baseline: 11 (18)  30 days: 2 (3.5)  Control  Baseline: 4 (6.8)  30 days: 6 (10.7) |
| Hammonds et al. [24] | Measured by dividing the actual number of pills taken by the expected number of pills taken during the study period and multiplying by 100. Adherent behaviour was defined as taking 80-100% of prescribed medication. | Rate of adherence, excluding over adherent users, is presented for control and intervention groups at end of follow-up. | Rate of adherence (SD) for participants who were not over users  Intervention  Baseline: not provided  30 days: 76.5 (20.9)  Control  Baseline: not provided  30 days: 70.4 (18.2) |
| Horvath et al. [30] | Measured by answering each of the following questions on a pull-down menu with responses that ranged from 0 to 100 in 1-point increments, (1) the percentage of time ART was correctly taken as prescribed in the past 30 days (2) the percentage of time ART was taken within 2 h of the scheduled dose in the past 30 days and (3) the percentage of time ART was taken while using stimulant drugs in the past 30 days. | Mean percentage of ART correctly taken as prescribed in past 30 days for control and intervention groups at baseline and end of follow-up. | Mean (SD)  Intervention  Baseline: 81.8 (25.4)  4 months: 89.0 (17.0)  Control  Baseline: 78.1 (29.6)  4 months: 77.2 (30.6) |
| Lakshminarayana et al. [31] | Measured by MMAS-8.^a^ | Mean MMAS-8 scores for control and intervention groups at baseline and end of follow-up. | Mean (SD)  Intervention  Baseline: 6.03 (1.57)  16 weeks: 6.3 (1.52)  Control  Baseline: 5.82 (1.48)  16 weeks: 5.74 (1.53) |
| Márquez Contreras et al. [27] | Measured by electronic monitoring devices in the lid of the drug container that automatically registers the time and date of opening. The number of times the lid was opened was measured, assuming that one pill was taken with each opening. The average adherence percentage was calculated by dividing the total number of pills assumed to have been taken by the total number of pills that should have been taken, multiplied by 100. A percentage between 80-100% was considered adherent and was calculated for global adherence (percentage of all doses taken in an established period), daily adherence (percentage of days on which drugs were taken correctly), correct time adherence (percentage of patients taking medication at the prescribed time) and therapeutic cover adherence (percentage of therapeutic coverage assuming a 24-hour therapeutic effect of antihypertensive drugs). | Global adherence percentages for control and intervention groups at end of follow-up. | % (95% CI)  Intervention  Baseline: not provided  12 months: 91.78 (85.48-98.08)  Control  Baseline: not provided  12 months: 62.66 (51.26-73.14) |
| Mira et al. [32] | Measured by the MMAS-4.^b^ | Mean MMAS-4 scores for control and intervention groups at baseline and end of follow-up. | Mean (SD)  Intervention  Baseline: 6.6 (1.2)  3 months: 7.4 (0.9)  Control  Baseline: 7.2 (0.9)  3 months: 7.3 (0.7) |
| Morawski et al. [25] | Measured by the MMAS-8. The possible scores ranged from 0 to 8, with lower scores indicating lower adherence. A score of less than 6 was classified as low adherence, from 6 to 7 was classified as moderate adherence and equal to 8 was classified as high adherence. | Mean MMAS-8 scores for control and intervention groups at baseline and end of follow-up. | Mean (SD)  Intervention  Baseline: 6.0 (1.8)  12 weeks: 6.3 (1.6)  Control  Baseline: 5.7 (1.8)  12 weeks: 5.7 (1.8) |
| Osahon et al. [33] | Measured by a category within a 31-item survey questionnaire that asked participants 5 questions related to medication adherence. Each answer was given one of the corresponding points: never (1), rarely (1/2), frequently (0), always (-1/2) and yes/no (-1). A summed score between 0 - 2 was classified as poorly adherent, 2.5 - 3.5 was classified as moderately adherent and 4 - 5 was classified as adherent. | Mean medication adherence score, calculated from the survey questionnaire, for control and intervention groups at end of follow-up. | Mean (SD)  Intervention  Baseline: not provided  2 months: 3.44 (1.52)  Control  Baseline: not provided  2 months: 3.24 (1.65) |
| Santo et al. [37] | Measured by the MMAS-8. Higher scores indicated higher medication adherence. | Mean MMAS-8 scores for control and intervention groups at baseline and end of follow-up. | Intervention  Baseline, Mean (SD): 6.8 (1.4)  3 months, Mean (95% CI): 7.11 (6.90 - 7.31)  Control  Baseline, Mean (SD): 7.0 (1.29)  3 months, Mean (95% CI): 6.63 (6.35 - 6.92) |
| Svendsen et al. [34] | Measured by an electronic monitor attached to the medication dispenser that registered the day and time patient used medication, an electronic balance at the clinic that weighed the medication canister and by patient self-reporting on a study specific, four-point ordinal scale. | Number and percentage of adherent patients to treatment sessions (defined as having applied foam treatment ≥ 80% of days in the treatment period) for control and intervention groups at end of follow-up. | N (%), 95% CI  Intervention  Baseline: not provided  4 weeks: 39 (65%), 53-77  Control  Baseline: not provided  4 weeks: 23 (38%), 26-51 |
| Teong et al. [35] | Measured by the MMAS-4. Each item was answered with a Yes (0) or No (1). The possible score ranged from 0 to 4. | Mean MMAS-4 scores for control and intervention groups at baseline and end of follow-up. | Mean (SD)  Intervention  Baseline: 2.1 (1.1)  12 weeks: 3.2 (1.1)  Control  Baseline: 2.1 (1.1)  12 weeks: 2.9 (1.3) |
| Mohammadi Torkabad et al. [36] | Measured by the MMAS-8. The scale had seven items with answers corresponding to Yes (0) and No (1) and a five-point option with answers corresponding to never/rarely, once in a while, sometimes, usually and all the time. A score of less than 6 was classified as poor medication adherence, from 6 to 8 was classified as moderate adherence and equal to 8 was classified as good adherence. | Median of MMAS-8 medication adherence scores for control and intervention groups at baseline and end of follow-up. | Median (IQR)  Intervention  Baseline: 3.7 (1.0)  3 months: 7.0 (1.0)  Control  Baseline: 3.5 (1.0)  3 months: 4.0 (1.0) |

^a^MMAS-8: 8-item Morisky Medication Adherence Scale

^b^MMAS-4: 4-item Morisky Medication Adherence Scale

### **References**

24. Hammonds T, Rickert K, Goldstein C, et al. Adherence to antidepressant medications: a randomized controlled trial of medication reminding in college students. J Am Coll Health. Apr 3, 2015;63(3):204-208. [doi: 10.1080/07448481.2014.975716] [Medline: 25338175]

25. Morawski K, Ghazinouri R, Krumme A, et al. Association of a smartphone application with medication adherence and blood pressure control: the MedISAFE-BP randomized clinical trial. JAMA Intern Med. Jun 1, 2018;178(6):802-809. [doi: 10.1001/jamainternmed.2018.0447] [Medline: 29710289]

26. Guhl E, Althouse AD, Pusateri AM, et al. The atrial fibrillation health literacy information technology trial: pilot trial of a mobile health app for atrial fibrillation. JMIR Cardio. Sep 4, 2020;4(1):e17162. [doi: 10.2196/17162] [Medline: 32886070]

27. Márquez Contreras E, Márquez Rivero S, Rodríguez García E, et al. Specific hypertension smartphone application to improve medication adherence in hypertension: a cluster-randomized trial. Curr Med Res Opin. Jan 2, 2019;35(1):167-173. [doi: 10.1080/03007995.2018.1549026] [Medline: 30431384]

28. Abu-El-Noor NI, Aljeesh YI, Bottcher B, Abu-El-Noor MK. Impact of a mobile phone app on adherence to treatment regimens among hypertensive patients: a randomised clinical trial study. Eur J Cardiovasc Nurs. Jun 29, 2021;20(5):428-435. [doi: 10.1177/1474515120938235] [Medline: 32631080]

29. Bozorgi A, Hosseini H, Eftekhar H, et al. The effect of the mobile “blood pressure management application” on hypertension self-management enhancement: a randomized controlled trial. Trials. Jun 24, 2021;22(1):413. [doi: 10.1186/s13063-021-05270-0] [Medline: 34167566]

30. Horvath KJ, Lammert S, MacLehose RF, Danh T, Baker JV, Carrico AW. A pilot study of a mobile app to support HIV antiretroviral therapy adherence among men who have sex with men who use stimulants. AIDS Behav. Nov 2019;23(11):3184-3198. [doi: 10.1007/s10461-019-02597-3] [Medline: 31309348]

31. Lakshminarayana R, Wang D, Burn D, et al. Using a smartphone-based self-management platform to support medication adherence and clinical consultation in Parkinson’s disease. NPJ Parkinsons Dis. 2017;3(1):2. [doi: 10.1038/s41531-016-0003-z] [Medline: 28649602]

32. Mira JJ, Navarro I, Botella F, et al. A Spanish pillbox app for elderly patients taking multiple medications: randomized controlled trial. J Med Internet Res. Apr 4, 2014;16(4):e99. [doi: 10.2196/jmir.3269] [Medline: 24705022]

33. Osahon PT, Mote LA, Ntaji VI. Assessment of the impact of medPlan®, a medication reminder mobile application, in glaucoma patients in Benin City, Nigeria. Trop J Pharm Res. 2021;19(12):2677-2682. [doi: 10.4314/tjpr.v19i12.28]

34. Svendsen MT, Andersen F, Andersen KH, et al. A smartphone application supporting patients with psoriasis improves adherence to topical treatment: a randomized controlled trial. Br J Dermatol. Nov 2018;179(5):1062-1071. [doi: 10.1111/bjd.16667] [Medline: 29654699]

35. Teong LF, Khor BH, Ng HM, et al. Effectiveness of a nutritional mobile application for management of hyperphosphatemia in patients on hemodialysis: a multicenter open-label randomized clinical trial. J Pers Med. Jun 12, 2022;12(6):961. [doi: 10.3390/jpm12060961] [Medline: 35743746]

36. Mohammadi Torkabad S, Negahban Bonabi T, Heidari S. Effectiveness of smartphone-based medication reminder application on medication adherence of patients with essential hypertension: A clinical trial study. J Nurs Midwifery Sci. 2020;7(4):219. [doi: 10.4103/JNMS.JNMS_16_20]

37. Santo K, Singleton A, Rogers K, et al. Medication reminder applications to improve adherence in coronary heart disease: a randomised clinical trial. Heart. Feb 2019;105(4):323-329. [doi: 10.1136/heartjnl-2018-313479]
